# Fast algorithms for computing sequence distances by exhaustive substring composition

**DOI:** 10.1186/1748-7188-3-13

**Published:** 2008-10-28

**Authors:** Alberto Apostolico, Olgert Denas

**Affiliations:** 1 Academia Nazionale dei Lincei, Rome, Italy; 2 Department of Information Engineering, Universitá di Padova, Padova, Italy; 3 College of Computing, Georgia Institute of Technology, Atlanta, Georgia, USA

## Abstract

The increasing throughput of sequencing raises growing needs for methods of sequence analysis and comparison on a genomic scale, notably, in connection with phylogenetic tree reconstruction. Such needs are hardly fulfilled by the more traditional measures of sequence similarity and distance, like string edit and gene rearrangement, due to a mixture of epistemological and computational problems. Alternative measures, based on the subword composition of sequences, have emerged in recent years and proved to be both fast and effective in a variety of tested cases. The common denominator of such measures is an underlying information theoretic notion of relative compressibility. Their viability depends critically on computational cost. The present paper describes as a paradigm the extension and efficient implementation of one of the methods in this class. The method is based on the comparison of the frequencies of all subwords in the two input sequences, where frequencies are suitably adjusted to take into account the statistical background.

## Background

Measuring the information content of finite sequences has been an intensely sought after and yet elusive goal, perhaps dating back to von Mises' pursuit of the notion of randomness [1]. Among prominent attempts at such a measure, one would find Brillouin's usage of Shannon's redundancy [2], and Kolmogorov's approach to information [3] which Lempel and Ziv specialized [4] to design practical and elegant data compression methods. Since every notion of information invokes naturally a germane one of *conditional *or *mutual *information, it becomes natural to base measures of similarity on the latter and hence ultimately on some kind of relative compressibility [5]. This angle of approach is eliciting a growing interest in computational molecular biology (see, e.g., [6-17]), thus contributing to a long tradition of mutual fascination [2,18]. The surge may be attributable primarily to the increasing availability of whole genomes and proteomes, that makes standard comparison and distance measures, such as those based on edit distances and gene rearrangement, either computationally unbearable, or scarcely significant, or both. In this paper we rely on the existing literature for significance and concentrate instead on aspects of computational efficiency. The specific distances we consider constitute an extension of the method of [13]. In that approach, each organism is represented by a *composition vector *the components of which correspond to the numbers of various (overlapping) *k*-peptides, for a fixed *k*, in all the translated amino acid sequences from an organism's genome. The numbers are modified by subtracting a statistical background to highlight the role of selective evolution. The subtraction procedure is based on a (*k*-2)-th order Markov prediction and therefore the minimum *k *is 3. For any fixed value of *k*, known string algorithms support the computation of the distance at the outset in time linear in the input size. In what follows, we expand on the approach based on the distribution of *k*-mers by including all the values of *k *in the count. In other words, we consider the vector composition distances involving the collection of *all *words of *any *length *k *up to *any *arbitrarily preset *maximum *length *K*. As it turns out, this can be done at no extra cost, hence still in linear time. This is optimal, since a lower bound for processing a sequence of size *L *is trivially Ω(*L*). Some interest, however, also comes from the fact that the number of *k*-mers grows exponentially with *k*, and the number of all distinct *k*-mers of length up to *K *that actually occur in the input may be quadratic in the length *L *of the host sequence when *K *≈ *L*. Therefore, we cannot afford to tally the contribution of each *k*-mer individually.

The distances we compute here below may be considered an extension also of the one in [14], in which phylogeny reconstruction on a genomic scale was based on the average length of common substrings. However, the linear time implementation of our measure is fairly more involved. The main difficulty is imposed by having to take predictions into account. In fact, albeit this may be surprising to the neophyte, the bare computation of shared string counts (e.g., multiply the frequencies with which each of the possibly Θ(*L*^2^) shared subword appears in either sequence and add up all these products), is trivially done in linear time, based on string data structures and basic properties that have been well understood for over thirty years (see, e.g., [19]).

## Methods

Let *S *be a sequence of length *L *and consider, for each word *w *[1..*k*] of a given length *k *in *S*, the expression [13]:

(1) $$a(w)=\{\begin{array}{cc} \frac{p(w[1..k])-p^{o}(w[1..k])}{p^{o}(w[1..k])} & \begin{array}{cc} \text{for} & p^{o}(w[1..k])\neq 0 \end{array}\\ 0 & \text{otherwise} \end{array}$$

where *p*(*w *[1..*k*]) is the observed ratio *f*(*w*)/(*L *- |*w*| + 1) between the count (possibly, zero) and the number of possible occurrences of the word *w *in *S*, and *p*^*o*^(*w *[1..*k*]) is the Markovian estimate of the probability *p *defined as

$$\frac{p(w[1..k-1])-p(w[2..k])}{p(w[2..k-1])}.$$

With easy passages the above can be rewritten as

(2) $$a(w)=\{\begin{array}{ll} \Lambda_{k}\times \frac{f(w[1..k])f(w[2..k-1])}{f(w[1..k-1])f(w[2..k])}-1 & \text{for}\\ \begin{array}{cc} f(w[1..k-1])\geq 1 & \text{and} \end{array} & f(w[2..k])\geq 1\\ 0 & \text{otherwise} \end{array}$$

Where

$$\Lambda_{k}=\frac{{\left(L-k+2\right)}^{2}}{\left(L-k+1)(L-k+3\right)},$$

so that the difference between the empirical probability of *w *and its Markov-based prediction, divided by the latter is represented by Expression 2 as well. For a given collection of words (e.g., the set of all *k*-mers for a fixed *k*), all *a*-values are stored, in some suitable order in a vector, called the *composition vector*. For two composition vectors *A *and *B*, the following distance function is considered:

(3) $$D(A,B)=1/2\left(1-\frac{\sum a_{i}b_{i}}{{\left(\sum a_{i}^{2}\times \sum b_{i}^{2}\right)}^{1/2}}\right)$$

where the *a*_*i*_'s and *b*_*i*_'s are computed by applying Expression 2 respectively to *A *and *B*.

There are |Σ|^*k *^components in the composition vector of *k*-mers, hence the size of these vectors grows exponentially with *k*, and so does the direct computation of *D*. It is clear that for any value of *k *the number of *k*-mers in a sequence of *L *characters is *O*(*L*). Less obvious but also well known (cf., e.g., [19]), is the fact that the *O*(*L*) bound applies as well to a notable class of words, defined as follows. A word is *maximal *in its host sequence if it is impossible to extend it by appending one or more characters without losing some of its occurrences. On the other hand, the total number of distinct words of any length found in a sequence of *L *characters can be Θ(*L*^2^).

In what follows, we show that it is possible to compute the measure *D *for composition vectors consisting of all (possibly Θ(*L*^2^)) words in the input sequences in overall time linear in the total length of the input. Our construction is supported by the basic structure of a suffix tree, which we proceed to recapture. In short, a *suffix tree *is a *trie *(i.e., a digital search index) collecting all suffixes of a string. For a compact representation, all chains of unary nodes are collapsed into a single arc, so that the resulting structure is linear in the length of the string. Whereas a string of *L *characters may contain Θ(*L*^2^) distinct substrings, the *O*(*L*) substrings terminating at branching nodes of the suffix tree are enough to represent the entire vocabulary of the string: for any string *w *not ending on a branching node, its shortest extension *w' *reaching such a branching node has exactly the same frequency (hence the same list of occurrences) as *w*. Hence these words are maximal in the sense described earlier. Unlike any straightforward implementation of this well known property, our construction must be based on normalized frequencies rather than bare counts, thereby implicating the Λ terms that do vary along every arc. One more level of complication stems from the fact that our computation needs access, for any word *w*, to the normalized frequencies of extensions of *w *in the form *aw*, with *a *a character of Σ, whereas such words might lack a branching node in the tree.

Suffix trees and their variants are ubiquitous data structures of string processing, and multiple algorithms are available for their construction in linear time and space. Our implementation is based on the *K*-truncated suffix tree [20], a special variant of the suffix tree that collects all subwords of length up to *K *instead of all suffixes of the sequence. This further reduces space and time costs in all cases where interest is limited to words of bounded length.

## Results and discussion

We now discuss adaptations of our trie for computing the compositional distance between two sequences according to Expression 3. It is convenient to subdivide the discussion into two parts, handling first the easier case of branching nodes, i.e., nodes that correspond to maximal words.

### Maximal Words

As part of the trie construction for either one of the sequences, each node *ν *is assigned the occurrence count of word ⟨*ν*⟩ in that sequence, where ⟨*ν*⟩ denotes the word spelled out by the labels found on the path from the root to the node *ν*. As is well known (cf., e.g., [19]), it is easy to update this information during each word insertion in the trie, if the latter is built by direct methods, or to compute it off-line (by attributing to each node the number of leaves in the subtree rooted at that node) when the suffix tree is built by one of the existing linear time constructions.

From inspection of *a*(*w*), it is seen that in order to compute probability estimates we actually need access, for any maximal word *w *[1..*k*] = ⟨*ν*⟩, to the occurrence counts of *w *[1..*k *- 1], *w *[2..*k*] and *w *[2..*k *- 1]. This is possible provided that for every node *ν *there is (1) a link from *ν *to *parent*(*ν*), where *parent*(*ν*) denotes the branching node on the root-ward path from *ν*, and (2) a *suffix *link from *ν *to *s*(*ν*) = $\hat{\nu }$ such that if ⟨*ν*⟩ = *aw *with *a *∈ Σ then ⟨$\hat{\nu }$⟩ = *w*. *At branching nodes*, both features are easily accommodated by the data structure, in fact, the second one is an essential part of any of its linear-time constructions. As we shall see, the computation of *D *is not entirely trivial when we take all subwords of *S *into account.

Imagine now that for two input sequences their respective tries are drawn each with a different color, and then superimposed. Only the words occurring in both sequences will contribute to the numerator in Expression 3. Such words are found on paths and nodes bearing both colors. On the other hand, words found on a path with only one color contribute to only one of the sums appearing in the denominator of 3. Finally, there are some words not appearing in one or both sequences that nevertheless contribute to Expression 3. Such words will be called *chimeral words*. With reference to one of the sequences, these are *k*-mers *w *such that *f*(*w *[1..*k*]) = 0, but *f*(*w *[1..*k *- 1]) ≥ 1 and *f*(*w *[2..*k*]) ≥ 1 in that sequence. The *a *value of these words is -1, and the words themselves would represent some of the possible unit-symbol extensions of paths that exist in the trie of the host sequence. Thus, for any word *w *[1..*k*], its contribution to the distance is to be accounted for only when *w *[1..*k *- 1] and *w *[2..*k*] both exist in the trie, but in no other case. The collection of these observations lead to reduce the number of words for which the components of Expression 2 need to be computed.

The computation of the second ratio of Expression 2 is easy to handle at branching nodes. To see this, consider one of the sequences and define the following function on each node *ν *in the trie associated with it.

(4) $$\Gamma (\nu )=\{\begin{array}{ccc} 1 & \text{if} & |e(\nu )|>1\\ \frac{f(\langle parent(\nu )\rangle )}{f(\langle \nu \rangle )} & \text{if} & |e(\nu )|=1 \end{array}$$

where |*e*(*ν*)| is the length of the label of the edge entering node *ν*. This function gives the occurrence count ratio between a node *ν *and its parent, and is straightforward to implement. Thus, substituting Expression 4 and Λ in the score 2 for each node *ν *we have

(5) $$Score(\nu )=\{\begin{array}{ccc} \Lambda \times \frac{\Gamma (s(\langle \nu \rangle ))}{\Gamma (\langle \nu \rangle )} & \text{for} & f(w[1..k])\geq 1\\ 0 & & \text{otherwise} \end{array}$$

where *s*(⟨*ν*⟩) is the proper longest suffix of *w *= ⟨*ν*⟩, that is, the word from the root to the node referenced by the suffix link that goes out from *ν*. The computation of the distance *D *simply requires to account separately for the frequency counts of either "color" in the generalized trie for the two input sequences. In summary, a procedure is readily set up for computing in linear time the contribution of all maximal words to the distance between sequences *S*_1 _and *S*_2_. The procedure builds a generalized suffix tree possibly truncated at some arbitrarily fixed length *K*. Each node of the trie contains information such as frequency, colors, edge length, and an id. The *Score *value (respectively, *a*(*w*) and *b*(*w*)) relative to *S*_1 _and *S*_2 _is computed at each node while *D*(*S*_1_, *S*_2_) is globally accumulated as the computation proceeds. This is further expanded at no extra cost to compute distances based on all shared maximal words, i.e., the words ending at branching nodes in the trie.

### Non-maximal Words

Recall that for any word *w *terminating in the middle of an arc, its shortest extension *w' *reaching a branching node has exactly the same frequency as *w*. We will show now that it is entirely feasible to include in the count also all such non-maximal words without stretching the time complexity to quadratic. Finally, we will show that the words that do not occur in the sequences, but whose prefixes and suffixes do, can also be handled without penalty. Combined with the preceding discussion, this will lead to the following

**Main Theorem ***The distance D resulting from the composition vectors relative to all words in two sequences can be computed in time and space linear in the input size*.

**Proof**. The claim will be established by exhibiting the completion of our construction.

We consider the combined trie for both sequences and discuss first how the contribution of all words that do appear in the trie (refer to Expression 2) is computed. As seen earlier in the discussion, this is easy for words ending precisely at a node. Let then *ν *be a node reached by an edge with a label of length *l *> 1, and let *ν*_1 _...*ν*_*l*-1 _be the unary nodes, numbered from *ν *toward the root, implicitly found on that edge. Let further *μ *be the branching node that is the parent of *ν *in the trie, $\hat{\nu }$ and $\hat{\mu }$ be nodes respectively reached by the suffix link from *ν *and *μ*, and make the simplifying assumption, to be later removed with no penalty, that there are no branching nodes between $\hat{\nu }$ and $\hat{\mu }$. The contribution of *ν*, *ν*_1_, ..., *ν*_*l*-1 _is the sum of:

• the contribution of *ν*, *ν*_1_, ..., *ν*_*l*-2_; zero if *l *= 2

• the contribution of *ν*_*l*-1_

The second component is to be handled in the standard way. As for the first component, under our assumption, each Γ in the ratio of Expression 5 gets the value 1, as does the ratio itself. Hence the first component increases the terms $\sum a_{i}b_{i}$, $\sum a_{i}^{2}$ and $\sum b_{i}^{2}$ respectively by:

(6) $$\sum_{k=2-l..0}\left(\Lambda_{k+depth(\nu )}-1\right)\times ({\Lambda^{\prime }}_{k+depth(\nu )}-1)$$

(7) $$\sum_{k=2-l..0}{\left(\Lambda_{k+depth(\nu )}-1\right)}^{2}$$

(8) $$\sum_{k=2-l..0}{\left({\Lambda^{\prime }}_{k+depth(\nu )}-1\right)}^{2}$$

where Λ_*k *_and ${\Lambda^{\prime }}_{k}$ denote the Λ function as applied to the first and second sequence, respectively, with varying *k*. We now introduce three vectors *X*, *F *and *S*, each of size equal to the maximum word length *K*, and with *l*th components (1 <*l *≤ *K*) respectively defined as:

$$X(l)=\sum_{i=1}^{l}\left(\Lambda_{i}-1)\times ({\Lambda^{\prime }}_{i}-1\right)$$

$$F(l)=\sum_{i=1}^{l}{\left(\Lambda_{i}-1\right)}^{2}$$

$$S(l)=\sum_{l}^{i=1}{\left({\Lambda^{\prime }}_{i}-1\right)}^{2}$$

We have then

(9) $$\sum_{k=2-l..0}\left(\Lambda_{k+depth(\nu )}-1)\times ({\Lambda^{\prime }}_{k+depth(\nu )}-1)=X[depth(\nu )]-X[depth(\nu )-l\right]$$

(10) $$\sum_{k=2-l..0}{\left(\Lambda_{k+depth(\nu )}-1\right)}^{2}=F[depth(\nu )]-F[depth(\nu )-l]$$

(11) $$\sum_{k=2-l..0}{\left({\Lambda^{\prime }}_{k+depth(\nu )}-1\right)}^{2}=S[depth(\nu )]-S[depth(\nu )-l]$$

where *depth*(*ν*) is the sum of the lengths of the labels on the path from the root to *ν*. The vectors *X*, *F S *can be computed once for all in time *O*(*K*) at the beginning of the execution since they depend only on *K*, *L*_1 _= |*S*_1_| and *L*_2 _= |*S*_2_|.

We claim now that removing the simplifying assumption that was made above is doable without penalty. As mentioned, the difficulty lies in the circumstance, that while every node *ν *with ⟨*ν*⟩ = *aw *has a suffix link defined to a node *μ *with ⟨*μ*⟩ = *w*, the converse is not necessarily true, i.e., there are nodes not reached by a suffix link for some or all of the characters of the alphabet. To handle this potential bottleneck, we introduce *dummy *unary nodes on each arc, in such a way that for any node *μ*, with ⟨*μ*⟩ = *w*, and *a *∈ Σ, if *aw *is a word of the input without a proper locus in the trie, then a dummy node *ν *such that ⟨*ν*⟩ = *aw *will be injected into the trie to mark that locus, and a suffix link will be issued from *ν *to *μ*. With dummy nodes in place, the restriction in the above construction is levied, in the sense that if *μ *is the (possibly dummy) node that is the parent of *ν *in the trie, and $\hat{\nu }$ and $\hat{\mu }$ are the nodes respectively reached through the suffix link from *ν *and *μ*, then there are no nodes between $\hat{\nu }$ and $\hat{\mu }$. The introduction of dummy nodes can be carried out in a post-processing of the trie that takes an overhead proportional to the overall number of nodes introduced. Consider each of the original arcs in the trie in some order. For each arc, following the suffix links from the terminal nodes identifies a path containing zero or more nodes, that can be scanned in time proportional to their number. Each such node invokes splitting of the arc under consideration by a dummy node, and the consequent setting of a suffix link to it. Knowing the length of the original arc label enables the identification of the split site and the subsequent relabeling of arcs. Thus all tasks are trivially accomplished in constant time. The number of dummy nodes inserted on account of any original node is bounded by the size of the alphabet, whence for finite alphabet this expansion of the trie takes linear time.

### Chimeral Words

So far in our discussion, we neglected all cases where *a*(*w*) = -1 (*f*(*w *[1..*k*]) = 0 in Case 1 of Expression 2). Such chimeral words take the form *w *= *avb*, *a*, *b *∈ Σ where *av *and *vb *occur in the input even though *w *does not. We can handle these words as part of the management of their infix *v*, thanks to the following easy property.

**Property 1 ***For any a ∈ Σ, if v does not end at a branching node then neither av does*.

This means that if *v *ends in the middle of an arc no work is needed: there cannot be any *vb *such that *av *and *vb *occur in the input while *w *does not! Hence *v *must end at a branching node, call it *ν*, and we are left with two cases, depending on whether *av *ends at a dummy or at an original branching node. In the first case, let *c *be the character following *av *on this path. We just need to add to the score the (-1) contribution of the branch of *ν *whose label begins by *b*. As is easily seen, every branch of *ν *except the one whose label begins by *c *similarly contribute at a rate of -1 each, whence subtracting one from the fan out of *ν *is all is needed to take into account all chimeral words induced by *av *and *vb *for some *b *∈ Σ. Finally, let *av *terminate at a branching node *μ*. Clearly, every branch of *μ *is replicated in a branch of *ν *whose label begins by the same character. The only chimeral words can originate from branches of *ν *that are not replicas of corresponding ones for *μ*. The bare count of such excess branches yields the contribution of all chimeral words implicated by *av *and some *vb*.

This concludes the computation of the distance based on *all *words common to two sequences of total length *L *in optimal *O*(*L*) time and space.

## Discussion

The various versions of the procedure have been implemented in combination with the PHYLIP's Neighbor-Joining package [21] and a web server has been predisposed for it at . An environment has been set up to carry out coordinated runs of experiments within each of the three main modes of operation described earlier. The first mode corresponds thus to distances involving only those common words of fixed length *k *that are found exactly at this depth on the frontier of the truncated trie. For any fixed *K *≥ 3, the second set builds trees based on distances that include all words of length 3 ≤ *k *≤ *K *ending at branching nodes in the truncated trie or at leaves of this trie that coincide with branching nodes of the full one. These latter words are interesting in that each one of them represents the longest extension of one of its own prefixes having the same occurrence count as that prefix (on a long edge, this makes the ratio *f*(⟨ *parent*(*ν*)⟩)/*f*(⟨*ν*⟩) = 1). Finally, the third mode builds trees derived from all subword distances for various maximum lengths. This set exposes the relationship of fixed-length versus all-subwords distances, as well as the influence of adding all subwords to the branching-node words. It thus enables one to study the influence on the inferred evolutionary trees of the distance computations based on different selections of word length and vocabulary composition. The analytical results obtained by any of these three methods are automatically given in input to Neighbor-Joining for tree construction and drawing.

By way of illustration, we report here classifications obtained for small sets consisting of 10 organisms under the three main settings, that correspond respectively to distances taking into account the composition of (1) only *k*-mers for a fixed value of *k*, (2) maximal *k*-mers for all values of *k *up to a fixed maximum value *K*, and (3) all *k*-mers of length *k *up to a fixed maximum value *K*.

**Figure 1:**
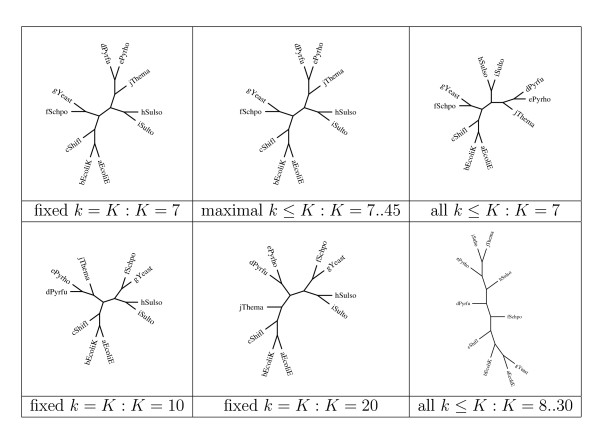
Phylogenetic trees derived for small samples under various compositional distances.

Figure 1 shows results obtained with a set of "distant" species, which would be presumed to be strongly separable and in fact they were. The dataset consists of:

**2 Eukaryotes **Schizosaccharomyces pombe (fSchpo) and Saccharomyces cerevisiae (gYeast)

**4 Archea **of which

• 2 Euryarchaeota: Pyrococcus furiosus (dPyrfu) and Pyrococcus hori-koshii (ePyrho)

• 2 Crenarchaeota: Sulfolobus solfataricus (hSulso) and Sulfolobus tokodaii (iSulto)

**4 Bacteria **of which

• 3 Proteobacteria: Escherichia coli O157:H7 EDL933 (aEcoliE), Escherichia coli K12 (bEcoliK) and Shigella exneri 2a str. 301 (cShifl)

• 1 Thermotogae: Thermotoga maritima (jThema)

The distance computations based on all *k*-mers is found to produce unreliable trees as soon as *K *> 7. At low level taxa, trees based on fixed-length *k*-mers and maximal *k*-mers are consistent, as they both correctly group together Eukaryotes, Proteobacteria, Euryarcheota and Crenarchaeota. However, at higher level taxa the distance based on maximal *k*-mers seems to be more stable. In fact, it groups Euryarcheota and Crenarchaeota in all cases, whereas with fixed-length *k*-mers this holds only for *K *≤ 9. All methods fail grouping Thermotogae with Proteobacteria, a deficiency that might be attributable to the absence of other organisms from the dataset.

Continuing with our illustration, we consider a sample of "similar" organisms, composed of:

**7 Firmicutes **of which

• Clostridium acetobutylicum ATCC824 (Cloab) and Clostridium perfringens (Clope)

• Streptococcus agalactiae NEM316 (StragN) and Streptococcus agalactiae 2603 V/R (StragV)

• Bacillus subtilis (Bacsu) and Bacillus anthracis str. Ames (Bacan)

• Thermoanaerobacter tengcongensis (Thete)

**1 Fuso **Fusobacterium nucleatum ATCC 25586 (Fusnu)

**1 Thermatogae **Thermotoga maritima (Thema)

**1 Aquificae **Aquifex aeolicus (Aquae)

**Figure 2:**
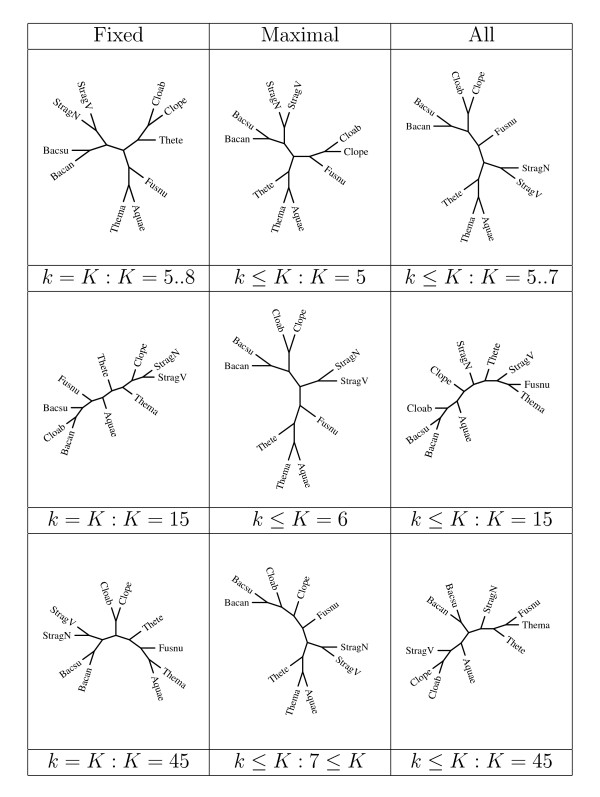
**Phylogenetic trees derived for small samples under various compositional distances.** With a sample of "closer" species, some differences appear in the tree already for small values of *k *(top row): all fixed *k*-mers with *k *= 5, same as for *k *= 6 or 7 (left); maximal *k*-mers up to a maximum *K *= 5 (center); and all *k*-mers up to a maximum *K *= 5, same as for *k *= 6 or 7 (right). This is repeated with *K *= 15 in the second row, with *K *= 45 in the third row except for the middle entry set at *K *= 6 and *K *= 7, respectively, since trees then stabilize for higher *K*.

Some of the corresponding trees are displayed in Figure 2. The distance based on fixed length *k*-mers behaves poorly even in the low taxa for *K *> 7 as it fails to group Cloab and Clope, Thema and Aquae, and so on. The trees based on maximal words remain stable both in high and low taxa as *K *increases, even though for *K *> 6 it fails to group Cloab and Clope. The trees based on all words diverge for *K *> 7. To summarize this and few other limited experiences, the distance based on fixed length *k*-mers seems to perform well for moderate values of *k*. For larger values of *k*, however, it seems to loose stability with "distant" organisms, and resolution with "close" ones. Somewhat surprisingly, the trees based on all *k*-mers also appear to be unstable with increasing *K*. On the other hand, the distance based on maximal words seems to produce consistent and stable trees. We stress that the purpose of our examples is only to illustrate the potential use and versatility of the tool. A thorough analysis of large data sets such as those that are becoming increasingly available falls well beyond the scope of the present paper.

## Conclusion

We presented fast and efficient tools for distance computations based on subword compositions as defined in [13]. This can be regarded as filling in part the gap between the rigid word length used in [13] and the shared-word length averaging of [14]. Our tools are also easily adapted to incorporate and subsume both of those approaches, thereby enabling the researcher to conduct a wide range of hypothesis testing on phylogeny and species relationships. The speedup achieved by such tools brings computations previously taking hours down to a couple of seconds. Our algorithms expand the roster of words that may partake in a distance measure, so as to include words of virtually unbounded length, thereby opening the way for the massive analysis of the future. By dithering with the three main modes of operation of our algorithm and the parameters *k *and *K*, it is possible to fine tune the selectivity and sensitivity of the method. The identification of the settings that are best suited to separate and classify each particular collection might be, per se, highly informative. Our tools can be deployed in the framework of phylogenetic tree reconstruction, but also in a much broader and growing spectrum of applications calling for subword analysis on a genomic scale.

## Note

A preliminary version of this paper formed the subject of a Keynote delivered at the IEEE Information Theory Workshop held in Porto, Portugal, on May 5–8, 2008.
